# A Systematic Review and Meta-Analysis of Single-Incision Laparoscopic Cholecystectomy Versus Conventional Four-Port Laparoscopic Cholecystectomy

**DOI:** 10.7759/cureus.32524

**Published:** 2022-12-14

**Authors:** Chirag Pereira, Shankar Gururaj

**Affiliations:** 1 General Surgery, Royal Lancaster Infirmary, Lancaster, GBR; 2 General Surgery, Father Muller Medical College and Hospital, Mangalore, IND

**Keywords:** single-port laparoscopy, post-operative pain, single incision laparoscopic cholecystectomy, laparoscopic cholecystectomy, silc

## Abstract

The present systematic review compares single-incision laparoscopic cholecystectomy (SILC) with conventional laparoscopic cholecystectomy (CLC) with the aim of assessing early postoperative pain and morbidity. The secondary outcomes assessed were the duration of surgery, length of hospital stay, and conversion to open surgery. A systematic search for medical records was conducted on PubMed, Embase, Medline, and the Cochrane Library. Meta-analysis was conducted using Review Manager 5.4. A total of 14 randomized control trials met the eligibility criteria, involving a total of 1762 patients. Early postoperative pain (four to six hours) (mean difference (MD): -0.86; 95%; confidence interval (CI): -1.16 to -0.55) showed significantly better results in the SILC group but showed no difference on the first or second postoperative day. There were significantly fewer complications (relative risk (RR): 1.7; 95%; CI: 1.16-2.50)* *recorded in the CLC group as compared to the SILC group. Operative time (MD: 19.66; 95% CI: 13.21-26.11) was significantly longer in the SILC group, while the duration of hospital stay (MD: -0.01; 95% CI: -0.28-0.26) and conversion to open surgery (RR: 0.99; 95% CI: 0.20-4.82) showed no significant difference. SILC had a significantly longer operative time and more complications as compared to CLC. However, it was associated with significantly lower early post-operative pain.

## Introduction and background

The gold standard treatment for benign gall bladder disease is laparoscopic cholecystectomy since it is associated with less postoperative pain, a shorter duration of hospital stay, and an early return to normal activity [1]. Conventional laparoscopic cholecystectomy (CLC) makes use of four ports, i.e., two 10 mm ports and two 5 mm ports. Alternatively, surgeons have also used a single 10 mm port with smaller working ports of about 3.5 mm [2].

Single-incision laparoscopic cholecystectomy (SILC) was first described by Navarra et al. [3] in 1997, who reported 30 cases with favorable outcomes. The main concerns about this technique are its feasibility and safety. The basic principle of laparoscopic surgery is the triangulation of instruments to allow for better ergonomic work [3]. In SILC, there is increased clashing of surgical instruments due to a lack of triangulation, and only a limited number of instruments can be used at a time.

The aim of the current systematic review is to evaluate post-operative pain and morbidity as primary outcomes between SILC and CLC. The secondary outcome measures evaluated are the duration of surgery, length of hospital stay, and conversion to open surgery.

## Review

A systematic review with meta-analysis was carried out to compare SILC with CLC for benign gall bladder disease. CLC makes use of two 10 mm ports and two 5 mm ports. Systematic reviews and meta-analyses were designed and reported according to Preferred Reporting Items for Systemic Reviews and Meta-Analyses (PRISMA) [4].

Search strategy

**Figure 1:**
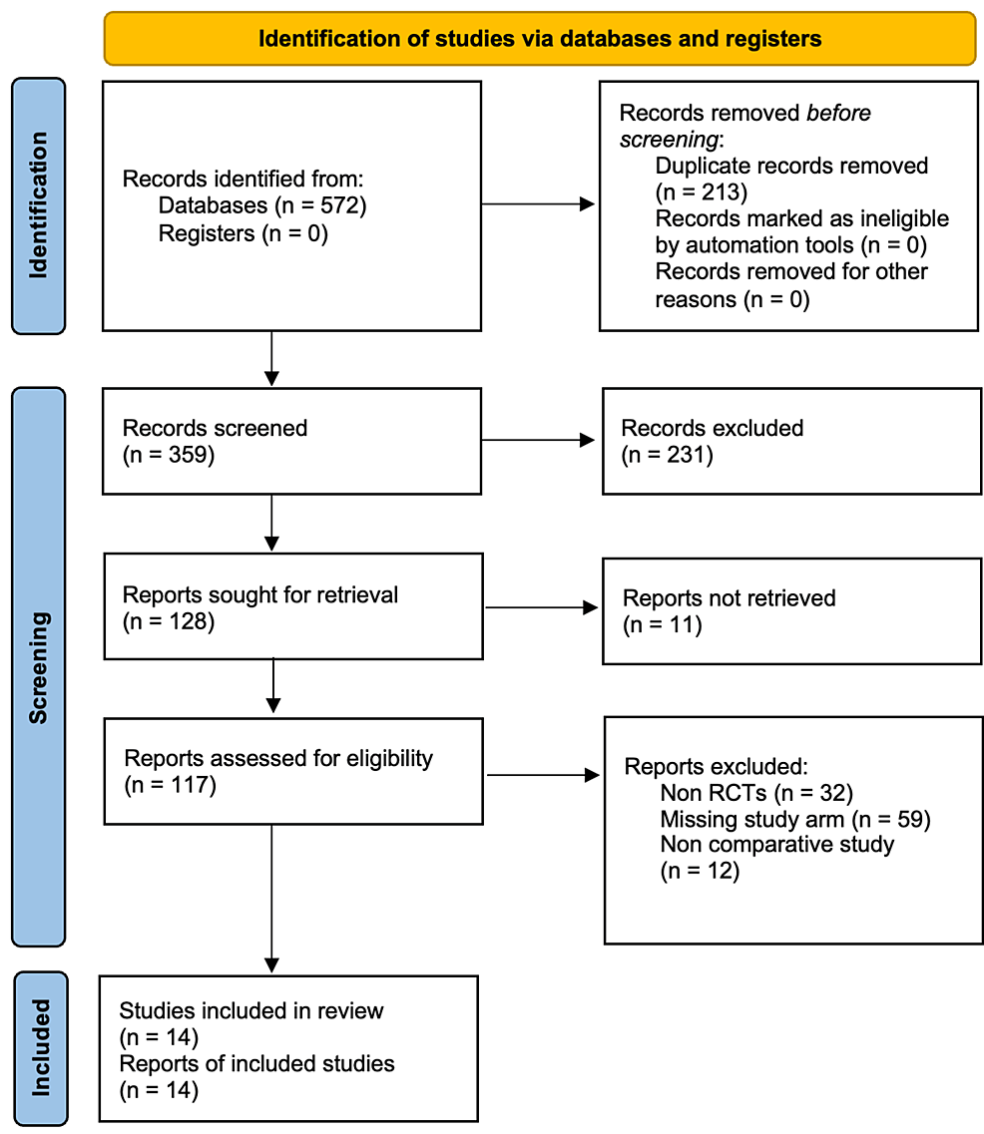
PRISMA flow diagram PRISMA: Preferred Reporting Items for Systemic Reviews and Meta-Analyses; RCTs: randomized control trials

Two authors conducted a comprehensive literature search from the Cochrane Library, PubMed, Embase, and Medline. The last date of the search was 6th, October 2022. Keywords used for electronic searches were "single incision," "SILC," "cholecystectomy," "laparoscopic cholecystectomy," "four-port," and "multi-port." Only human studies were included, and there was no publication date restriction. The PRISMA flow diagram of included studies is shown in Figure 1.

Study selection

Studies included for review were (1) randomized control trials (RCTs), (2) studies comparing SILC with CLC, and (3) appropriate information on outcome measures. Studies excluded were (1) non-RCTs; (2) studies comparing SILC with three-port cholecystectomy or miniport cholecystectomy; and (3) laparoscopic cholecystectomy (LC) performed with one 10 mm port and three 5 mm ports.

Data collection and assessment of the risk of bias

**Figure 2:**
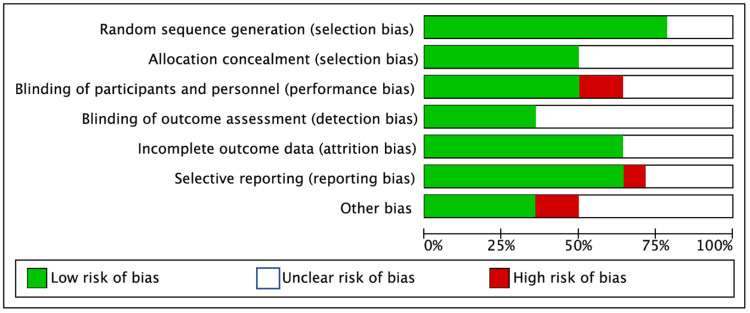
Risk of bias

Data were collected independently by two authors and included the first author, year and country of origin, study size, age, gender distribution, postoperative pain, morbidity, conversion to open surgery, duration of surgery, and duration of hospital stay. The Cochrane risk of bias tool [5] was used to assess the risk of bias for all included studies by two authors. The following categories were classified as low, high, or unclear: random sequence generation, allocation concealment, blinding of outcome assessment, blinding of participants and personnel, selective reporting, and other sources of bias (Figure 2).

Statistical analysis

Statistical analysis was performed using Review Manager 5.4. The risk ratio at a 95% confidence interval was calculated for dichotomous variables, and the mean difference was calculated for continuous data at 95% confidence intervals. The Cochrane Q test and the I2 test were used to assess heterogeneity in the included studies. 0% was considered no heterogeneity, while >50% was considered significant heterogeneity. Based on the calculated heterogeneity, random effect models and fixed effect models were used for analysis. In studies where the standard deviation was not recorded, it was estimated from the median and range [6].

Results

Fourteen RCTs were included in this systematic review, which comprised a total of 1762 patients. There were 747 patients in the SILC group and 1015 patients in the CLC group. Common exclusion criteria among the included studies were previous upper abdominal surgeries and acute cholecystitis. Six studies used computer-generated randomization, four used sealed envelopes, two used random number tables, and two did not mention the method of randomization. Patient characteristics for SILC and CRC are shown in Table 1 and Table 2, respectively.

**Table 1:** Patient characteristics in the SILC group SILC: single-incision laparoscopic cholecystectomy; BMI: body mass index

Study	Country (year)	Method of randomization	Study size	Age (years) mean ± SD	Sex (male/female)	BMI (kg/m^2^) mean ± SD
Lirici et al. [7]	Italy (2011)	Sealed envelope	20	45	6/14	25
Bucher et a.l [8]	Switzerland (2011)	Randomization table	75	45.7 ± 18.1	Not recorded	27.2 ± 3.7
Sinan et al. [9]	Turkey (2012)	Computer generated	17	48.5 ± 8.9	4/13	27.3 ± 3.1
Saad et al. [10]	Germany (2013)	Computer generated	35	45 ± 17	26/9	25.4 ± 3.1
Luna et al. [11]	Brazil (2013)	Not recorded	20	Not recorded	Not recorded	Not recorded
Abd Ellatif et al. [12]	Egypt (2013)	Sealed envelope	125	47.7 ± 10.6	20/95	26.9 ± 5.5
Jorgensen et al. [13]	Denmark (2014)	Computer generated	60	44.8 ± 5.6	0/60	26.3 ± 1.3
Sulu et al. [14]	Turkey (2015)	Not recorded	30	48.53 ± 7.4	9/21	30.3 ± 4.29
Lurje et al. [15]	Switzerland (2015)	Computer generated	48	48 ± 16	15/33	25 ± 3
Chang et al. [16]	Singapore (2015)	Sealed envelope	50	48.16 ± 12.53	19/31	25.34 ± 4.5
Guo et al. [17]	China (2015)	Sealed envelope	138	42.65 ± 11.86	33/105	24.68 ± 2.20
Partelli et al. [18]	Italy (2015)	Computer generated	30	51.25 ± 13.8	8/22	24.5 ± 4.3
Zhao et al. [19]	China (2016)	Random number table	50	48.7 ± 8.1	19/31	25.8 ± 3.2
Qu et al. [20]	China (2019)	Computer generated	49	44.63 ± 10.19	20/29	23.02 ± 2.60

**Table 2:** Patient characteristics in the CLC group CLC: conventional laparoscopic cholecystectomy; BMI: body mass index

Study	Country (year)	Method of randomization	Study size	Age (years) mean ± SD	Sex (male/female)	BMI (kg/m^2^) mean ± SD
Lirici et al. [7]	Italy (2011)	Sealed envelopes	20	50	6/14	27
Bucher et al. [8]	Switzerland (2011)	Randomization table	75	46.5 ± 16.7	Not recorded	25.7 ± 4.3
Sinan et al. [9]	Turkey (2012)	Computer generated	17	48.7 ± 14.3	8/9	27.2 ± 2.9
Saad et al. [10]	Germany (2013)	Computer generated	35	49 ± 14	26/9	25.4 ± 2.5
Luna et al. [11]	Brazil (2013)	Not recorded	20	Not recorded	Not recorded	Not recorded
Abd Ellatif et al. [12]	Egypt (2013)	Sealed envelope	125	46.9 ± 11.4	37/88	29.5 ± 5.6
Jorgensen et al. [13]	Denmark (2014)	Computer generated	60	45.2 ± 6.6	0/60	24.9 ± 1.8
Sulu et al. [14]	Turkey (2015)	Not recorded	30	44.04 ± 11.3	12/18	28.54 ± 5.5
Lurje et al. [15]	Switzerland (2015)	Computer generated	48	44 ± 13	19/29	26 ± 5
Chang et al. [16]	Singapore (2015)	Sealed envelope	50	52.34 ± 13.12	20/30	25.83 ± 6.4
Guo et al. [17]	China (2015)	Sealed envelope	414	44.44 ± 12.20	104/310	25.13 ± 2.96
Partelli et al. [18]	Italy (2015)	Computer generated	29	50.25 ± 13.5	14/15	24.5 ± 4.3
Zhao et al. [19]	China (2016)	Random number table	50	48.4 ± 9.2	14/36	24.7 ± 3.9
Qu et al. [20]	China (2019)	Computer generated	42	48.62 ± 8.88	21/21	23.74 ± 2.66

Postoperative pain and morbidity

**Figure 3:**
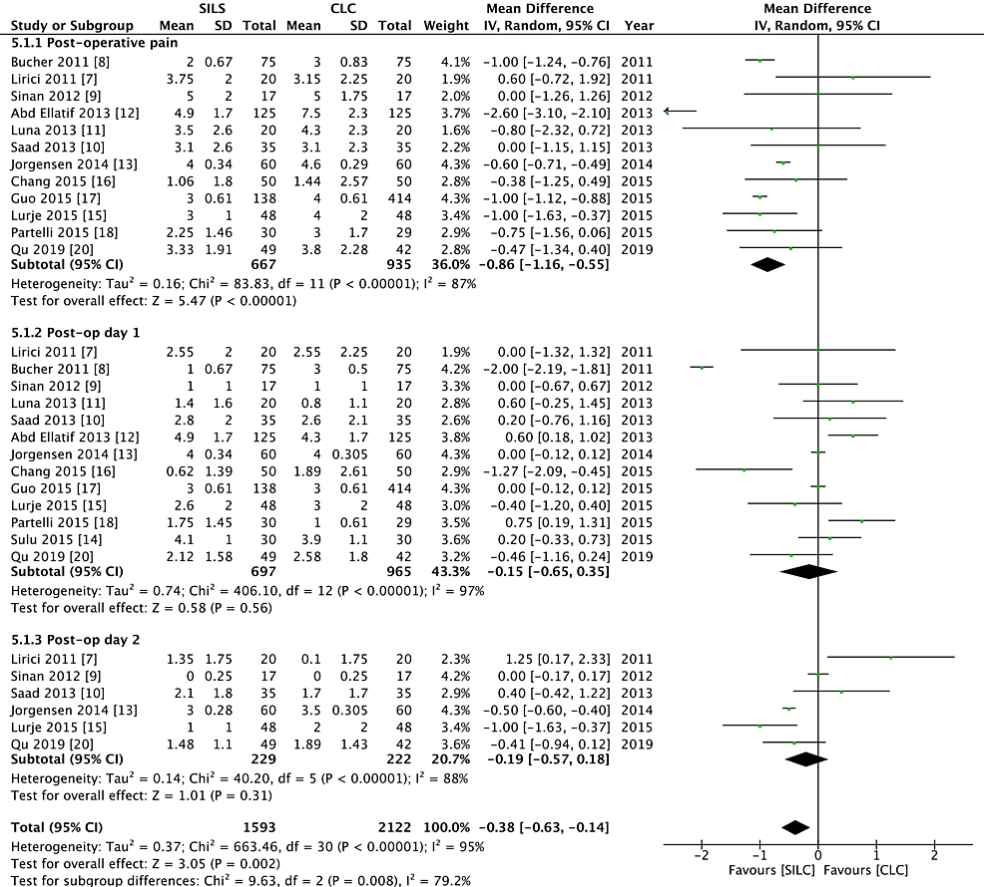
Forest plot of early post-operative pain SILC: single-incision laparoscopic cholecystectomy; CLC: conventional laparoscopic cholecystectomy

The primary outcome measures compared between the SILC and CLC groups were postoperative pain and overall morbidity. The pain was assessed using the visual analog scale (VAS). The immediate postoperative pain was assessed between four and six hours following the completion of surgery; this was reported by 13 RCTs, and it was found that postoperative pain was significantly lower in the SILC group. Pain assessed on the first and second postoperative days showed no significant difference between the two groups (Figure 3). The level of heterogeneity was high (I2 = 79.2%).

**Figure 4:**
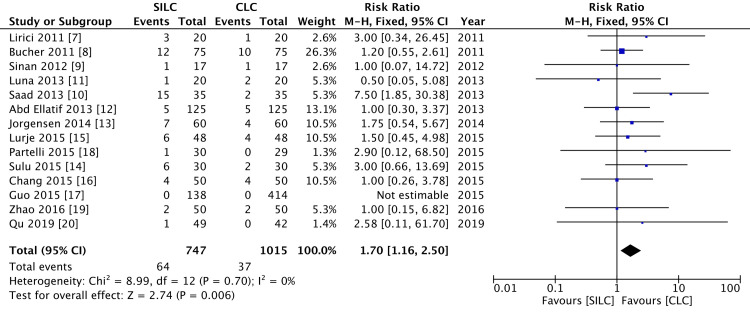
A forest plot of postoperative complications SILC: single-incision laparoscopic cholecystectomy; CLC: conventional laparoscopic cholecystectomy

Major adverse outcome measures assessed in these studies were bile duct injury, bile leak, and large intra-abdominal collections, while minor outcome measures were surgical site wound infection and small intra-abdominal collections managed conservatively. There were fewer complications recorded in the CLC group, which was significant, and the level of heterogeneity was low (Figure 4).

Secondary outcome measures between the SILC and CLC groups are shown in Table 3 and Table 4, respectively.

**Table 3:** Secondary outcomes in the SILC group SILC: single-incision laparoscopic cholecystectomy

Study	Operative time (min) Mean ± SD	Duration of hospital stay (days) Mean ± SD	Conversion to open surgery
Lirici et al. [7]	76.75 ± 17.5	2.5 ± 1.25	0
Bucher et al. [8]	66 ± 12.8	0 ± 0.33	0
Sinan et al. [9]	124.4 ± 29.7	Not recorded	Not recorded
Saad et al. [10]	45.7 ± 10.9	3.1 ± 0.6	0
Luna et al. [11]	92 ± 27.7	Not recorded	Not recorded
Abd Ellatif et al. [12]	62.7 ± 10.2	2.7 ± 0.5	0
Jorgensen et al. [13]	72.5 ± 8.75	Not recorded	Not recorded
Sulu et al. [14]	83 ± 40.4	1.96 ± 1	Not recorded
Lurje et al. [15]	101 ± 36	2 ± 0.25	1
Chang et al. [16]	79.46 ± 7.10	Not recorded	0
Guo et al. [17]	58.97 ± 21.56	3.5 ± 0.57	Not recorded
Partelli et al. [18]	67.5 ± 23.11	4.75 ± 3.7	1
Zhao et al. [19]	37.5 ± 7.21	Not recorded	Not recorded
Qu et al. [20]	46.89 ± 10.03	1.02 ± 0.14	0

**Table 4:** Secondary outcomes in the CLC group CLC: conventional laparoscopic cholecystectomy

Study	Operative time (min) Mean ± SD	Duration of hospital stay (days) Mean ± SD	Conversion to open surgery
Lirici et al. [7]	48.25 ± 66.25	2.65 ± 1.75	1
Bucher et al [8]	64 ± 13.2	1 ± 0.83	0
Sinan et al. [9]	64.1 ± 26.1	Not recorded	Not recorded
Saad et al. [10]	35 ± 14	3 ± 0.2	0
Luna et al. [11]	41.9 ± 14	Not recorded	Not recorded
Abd Ellatif et al. [12]	55.3 ± 8.9	2.4 ± 0.8	0
Jorgensen et al. [13]	40 ± 4.33	Not recorded	Not recorded
Sulu et al. [14]	65.8 ± 32.1	1.56 ± 0.8	Not recorded
Lurje et al. [15]	90 ± 41	2 ± 0.25	1
Chang et al. [16]	58.88 ± 34.06	Not recorded	0
Guo et al. [17]	43.38 ± 19.02	3.5 ± 0.57	Not recorded
Partelli et al. [18]	45 ± 11.56	2.75 ± 1.4	0
Zhao et al. [19]	23.75 ± 1.39	Not recorded	Not recorded
Qu et al. [20]	37.24 ± 10.23	1.16 ± 0.92	0

Operative time

**Figure 5:**
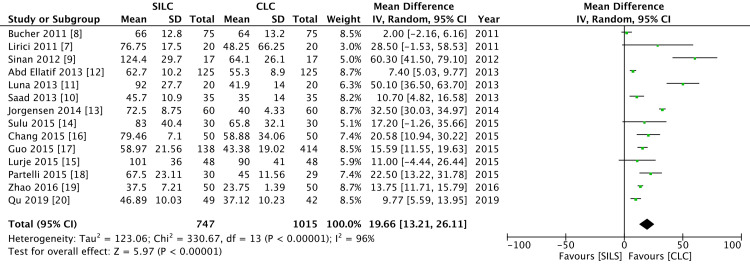
A forest plot of operative time SILC: single-incision laparoscopic cholecystectomy; CLC: conventional laparoscopic cholecystectomy

All 14 RCTs reported the same operative time. The operative time was significantly higher in the SILC group as compared to the CLC group. There was a high level of heterogeneity (I2 = 96%) (Figure 5).

Duration of hospital stay

**Figure 6:**
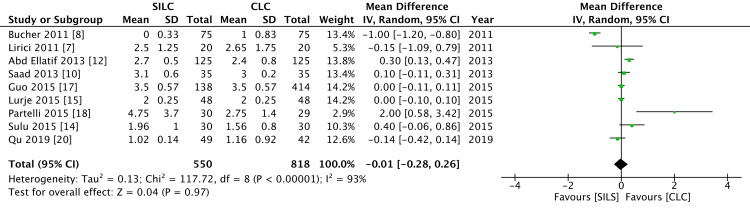
A forest plot of the duration of the hospital stay SILC: single-incision laparoscopic cholecystectomy; CLC: conventional laparoscopic cholecystectomy

Nine RCTs reported the duration of the hospital stay. There was no significant difference between the two groups (MD = -0.01; 95% CI = -0.28 to 0.26). There was a high level of heterogeneity (I2 = 93%) (Figure 6).

Conversion to open surgery

**Figure 7:**
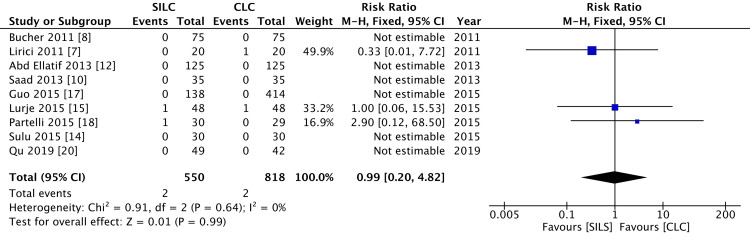
A Forest plot of conversion to open surgery SILC: single-incision of laparoscopic surgery; CLC: conventional laparoscopic surgery

Conversion to open surgery was reported by eight RCTs. There was no significant difference between the two groups (RR 0.99; 95% CI 0.20-4.82), and the level of heterogeneity was low (I2=0%) (Figure 7).

Discussion

The current systematic review included 14 RCTs consisting of a total of 747 patients in the SILC group and 1015 patients in the CLC group. In order to reduce inevitable bias, we only included RCTs in our review. There was a significant level of heterogeneity, as demonstrated by the analysis. SILC proved to be advantageous in terms of postoperative pain but was associated with more complications in comparison to CLC.

In the included studies, we looked at certain minor adverse outcomes such as wound infection and a small abdominal collection, and serious adverse outcomes such as bile duct injury, bile leak, and large intraabdominal collections in assessing the overall morbidity between the two groups. Based on our analysis, CLC was found to have a lower number of overall complications as compared to SILC. Evers et al. [21] conducted a systematic review that separated adverse outcomes into minor and serious adverse outcomes. They found that both minor and serious adverse outcomes were more common in SILC, similar to our study. Another review by Arezzo et al. [22] published earlier in 2013 reported a greater number of complications in the SILC group but found no significant difference between the two groups. There was no reported mortality in any of the studies.

The current review demonstrated that early postoperative pain (four to six hours following surgery) based on the VAS scale was significantly lower in the SILC group. We also evaluated post-operative pain on the first and second postoperative days but failed to show any significant difference. Our review mainly focused on assessing early post-operative pain in the two groups, as only a few RCTs assessed pain in the one- to two-week period following surgery. Evers et al. [21] assessed post-operative pain on the seventh postoperative day but failed to show any statistically significant difference between the two groups. The type of analgesia used and blinding protocols are not mentioned in all the included studies, and this will contribute to important sources of bias.

Operative time was significantly longer in the SILC group. This was consistent with previously published reviews [21, 22]. There is a longer learning curve in performing SILS as compared to CLC, which is due to the different types of single access ports available as well as the various instruments used, ranging from pre-curved to straight laparoscopic instruments [23]. There was no significant difference in regard to the duration of hospitalization or conversion to open surgery between the SILC and CLC groups.

Limitations

Limitations related to included studies include a lack of information about randomization, a lack of blinding, and incomplete or unclear data outcomes. Few studies had long-term follow-ups, hence assessing the late complications is not possible. Most studies included patients with a mean BMI of 25 kg/m2. Hence, this review fails to assess patients with a higher BMI, which is always more technically difficult to perform [24]. Analysis of the quality of life and operative cost factor was not performed because there was insufficient data or the evaluation varied significantly between studies. 

## Conclusions

SILC was associated with significantly lower postoperative pain in the immediate postoperative period but did not show any significant difference in the first or second postoperative days. There were a greater number of complications in the SILC group, and it took longer to perform as compared to the CLC group. We recommend that a well-structured, double-blind RCT with a clear description of the postoperative pain protocol and long follow-up periods be carried out to better assess the safety profile of SILC.
